# Current understanding of the genetics of thoracic aortic disease

**DOI:** 10.20517/2574-1209.2023.55

**Published:** 2024-01-21

**Authors:** Lauren E. Levy, Megan Zak, Jason P. Glotzbach

**Affiliations:** Division of Cardiothoracic Surgery, Department of Surgery, University of Utah School of Medicine, Salt Lake City, UT 84106, USA.

**Keywords:** Thoracic aortic disease, genomics, thoracic aortic aneurysm, thoracic aortic dissection

## Abstract

Thoracic aortic dissection is a feared, highly lethal condition most commonly developing from aneurysmal dilation of the thoracic aorta. Elective prophylactic replacement of thoracic aortic aneurysms dramatically mitigates this risk. However, diagnosis of a thoracic aortic aneurysm can be challenging. Thoracic aortic disease - horacic aortic aneurysm and dissection (TAAD) - can be sporadic or heritable. Patients with syndromic heritable TAAD present with classic phenotype and clinical features correlating to their disease. In contrast, patients with non-syndromic heritable disease are harder to diagnose due to their lack of defining uniform phenotypes. Recent advances in genomics have begun to elucidate the genetic underpinnings of non-syndromic TAAD (ns-TAAD) for better understanding this complex disease and improve diagnosis and management. Herein, we review the foundation of knowledge in ns-TAAD heritability and key research studies identifying gene mutations in vascular smooth muscle cells, the extracellular matrix, and TGF-beta signaling present in ns-TAAD. We summarize the current guidelines for the diagnosis, screening, and surgical management of ns-TAAD including recommendations for genetic testing of high-risk individuals. Finally, we highlight areas of future research that will continue to advance our understanding of the complex genetic and epigenetic factors in TAAD.

## INTRODUCTION

Thoracic aortic dissection is a feared, highly lethal condition that can occur sporadically or with a heritable presentation. Pre-hospital mortality can reach up to 61%, and approximately 50% of patients do not survive past 30 days of the index event^[1,2]^. Dissection and rupture are secondary to aneurysmal dilation of the thoracic aorta and are largely preventable with prophylactic thoracic aneurysm repair. While emergent perioperative repair mortality is approximately 20%^[3]^, perioperative mortality risk for elective repair is 3%^[4]^. The challenge herein is identifying those with asymptomatic aneurysms for elective repair. The most important risk factor for the development of thoracic aneurysm and dissection (TAAD) is a positive family history. Aortic aneurysms can be genetically triggered (hereditary) or sporadic (no clear genetic or familial cause). Hereditary TAAD is categorized as syndromic or non-syndromic. Syndromic aneurysms are associated with Mendelian inheritance patterns of known genetic mutations and a pathognomonic constellation of extra-thoracic features. Non-syndromic TAAD (ns-TAAD) may display a familial pattern, but their genetic mutations do not follow classic Mendelian inheritance patterns, nor do patients exhibit characteristic extra-thoracic features, which makes ns-TAAD significantly harder to recognize, especially if surveillance and follow-up within families are limited. Thoracic aortic aneurysms are typically clinically silent, and many are diagnosed either as incidental findings on imaging studies obtained for other reasons or when they develop into an acute aortic dissection. Specific guidelines for intervention in ns-TAAD are largely inferred from established syndromic TAAD and general population guidelines and are not yet nuanced to the spectrum of ns-TAAD genotypes and phenotypes^[5,6]^. To successfully evolve treatment paradigms, further quantification of heritability risk and a more thorough understanding of the details of genetic predisposition for ns-TAAD are needed. This article will review the current knowledge of the genetics of aortic disease, review management guidelines, and identify research opportunities around familial risk stratification in ns-TAAD.

## METHODS

We conducted a literature search using PubMed and Ovid search engines and applied no limitations on the date of publication. The included articles were published from 1996 to May 2023. Search terms used were thoracic aortic AND (aneurysm OR dissection), familial thoracic aortic AND (aneurysm OR dissection), non-syndromic thoracic AND (aneurysm OR dissection), hereditary thoracic aortic AND (aneurysm OR dissection), hereditary OR genetic AND (thoracic aortopathy), family AND (screening OR history) AND (thoracic aortic dissection OR thoracic aortic aneurysm), autopsy OR postmortem AND (thoracic aortic dissection OR thoracic aortic aneurysm), risk factor AND aortic AND (aneurysm OR dissection), aortic AND (aneurysm OR dissection) AND (sporadic OR familial), bicuspid AND aortic valve AND (aneurysm OR dissection), familial OR genetic (AND bicuspid aortic valve). Only publications in the English language were reviewed. Published guidelines from the American College of Cardiology Foundation/American Heart Association, Society of Thoracic Surgeons, American Association for Thoracic Surgery, European Society of Cardiology, and Canadian Cardiovascular Society were reviewed. Additionally, a manual search of references from publications was conducted. From these references, we chose pertinent topics: novel genes identified in the development of ns-TAAD, comparison of mortality risks between syndromic and ns-TAAD, histopathology of thoracic aortopathy, genetics of bicuspid aortic valve, and an expert consensus statement of screening guidelines.

## RESULTS

### Inheritance patterns

There are two types of hereditary thoracic aortopathies: syndromic and non-syndromic. Syndromic thoracic aortopathies, including Marfan syndrome (MFS), Loeys-Dietz syndrome (LDS), and vascular Ehlers-Danlos syndrome (vEDS), are associated with extra-thoracic connective tissue defects and are beyond the scope of this article. In contrast, ns-TAAD is isolated to the aorta with no associated syndromic defects^[7]^. Approximately 5% of patients presenting with TAAD have the disease due to syndromic aortopathies, implying that 95% are due to ns-TAAD or are sporadic^[8]^. Although much has yet to be investigated through pedigree and gene sequencing analysis, current data suggests that up to 20% of TAADs not due to MFS, LDS, or vEDS have a familial component, i.e., they are ns-TAAD^[9]^. The myriad of potentially pathologic genetic variants underlying ns-TAAD are only just beginning to be elucidated.

#### Postmortem studies

A true incidence of acute aortic dissection remains unknown as many of those affected do not survive to receive intervention and treatment. Tanaka *et al.* conducted a retrospective study of 311 patients who succumbed to out-of-hospital cardiopulmonary arrests and found 7% of those patients experienced acute type A aortic dissections^[1]^. A systematic review by Lovatt *et al*. analyzing 12 studies including 1,663 patients estimated the overall rate of misdiagnosis of aortic dissection to be 33.8%^[10]^. These findings suggest an underestimation of the prevalence of fatal dissections, specifically in patients presenting with chest pain, back pain, and syncope attributed to acute coronary syndrome, stroke, or pulmonary embolism and/or the absence of “typical features” of acute aortic dissection such as widened mediastinum on chest X-ray. A more recent multi-institutional autopsy study of acute dissection fatalities in 35 patients, all under the age of 35 years, aimed to stratify risk factors for dissection. Forty-three percent were associated with bicuspid aortic valve (BAV), followed by 31% with no valvulopathy or syndromic diagnosis association. Notably, 16 (47%) patients experiencing prodromal symptoms (e.g., chest pain, malaise, nausea, back pain) were misdiagnosed by a physician as having anxiety, gastroenteritis, esophageal reflux or spasm, pneumonia, or pleuritic pain^[11]^.

Milewicz *et al.* reviewed autopsy reports in conjunction with medical records in six families with multiple members affected with TAAD. They found that the inheritance patterns were consistent with an autosomal dominant pattern but also displayed variable penetrance, resulting in difficulty in accurately identifying at-risk individuals. In addition, two of these families did not have an identifiable known gene mutation^[12]^.

#### Pedigree analyses

A familial pattern in ns-TAAD was first identified in 1997 using a pedigree analysis of first-degree relatives of 158 affected probands. The study demonstrated a familial aggregation pattern but no clear Mendelian pattern of inheritance. Additionally, first-degree relatives (FDRs) of probands with thoracic aortic dissection were found to have a three-fold higher risk of sudden death^[13]^. Several familial studies have documented the heterogeneous nature of the inheritance pattern associated with ns-TAAD. Coady *et al.* performed a pedigree analysis from 135 probands and found variable patterns of inheritance with 38% as autosomal dominant, 27% as autosomal recessive, and 23% as X-linked dominant or autosomal dominant, supporting the idea of genetic heterogeneity in familial ns-TAAD^[14]^. These findings are corroborated by other groups. Albornoz *et al*. investigated pedigrees of 520 patients presenting with TAAD^[9]^. Twenty-one percent exhibited a familial pattern, with 77% following an autosomal dominant inheritance pattern with variable phenotypic expression. Studies over the past two decades by Milewicz *et al.* have also revealed that up to 20% of ns-TAAD is typically autosomal dominant, with decreased penetrance indicating the presence of heterozygous pathogenic variants^[13,15]^.

There is accumulating evidence that familial risk for acute aortic pathology confers an increased mortality risk for relatives of patients with thoracic aortic disease. A recent pedigree analysis of 100 probands found that the annual probability of developing an acute dissection was 2.77 times higher in FDRs of affected individuals compared to those without a family history of aortic dissection. They also found time to exposure was significantly decreased in FDR compared to those without a family history (18.3 *vs.* 43.1 years)^[16]^. We recently demonstrated a significant increase in cumulative lifelong aortic mortality in close relatives of patients with thoracic aortic disease using the Utah Population database^[17]^.

It is likely that this increased mortality risk is related to more aggressive rates of growth and aneurysm progression in the setting of ns-TAAD patients. Coady and Albornoz found an increased growth rate of aneurysms in the familial cases compared to sporadic and MFS: 0.21–0.22 cm/year *vs*. 0.03–0.16 cm/year and 0.10 cm/year, respectively^[9,14]^. Albornoz also found that familial aortopathy patients tended to be younger at the time of diagnosis compared to those with sporadic aneurysms. In Ma’s pedigree analysis of 51 sporadic dissections and 39 familial dissections, over half of the familial dissections presented within 10 years of the age at which their respective family member experienced dissection and an overall younger age than sporadic cases. Altogether, this data suggests an important role for screening in ns-TAAD that may inform earlier timing of prophylactic elective thoracic aortic aneurysm surgery to prevent dissection in younger patients with positive family history, especially if they are within 10 years of the age of onset in an affected relative^[18]^.

#### Observational studies

Several observational and database studies have focused on elucidating risk factors, incidence, and outcomes of TAAD. The International Registry of Acute Aortic Dissection (IRAD) was established in 1996 to collect data on demographics, history of present illness, physical exam, imaging, management, and outcomes across over 50 centers worldwide to better understand acute aortic pathology. Januzzi *et al.* reviewed IRAD data of 951 patients with aortic dissection to investigate risk factors and mortality. Patients aged 40 years and younger were found to have larger aortic diameters and less likely to have hypertension. Of these younger patients, 69% had either MFS or prior aortic valvulopathy, but 31% of patients had no known genetic etiology or aortic valvular disease. Mortality rates were unexpectedly similar between the older and younger groups and were noted not to be due to a delay in diagnosis^[19]^. In 2018, Evangelista *et al.* reported on the IRAD 20-year experience, noting that 60% of patients presenting with acute aortic dissection had maximum aortic diameters less than 5.5 cm - the guideline-recommended diameter for elective thoracic aortic aneurysm repair - and 40% actually had a diameter less than 5 cm - a cohort that included more patients with MFS and BAV^[20]^.

In a Swedish population-based study of over 14,000 patients, the incidence of thoracic aortic disease was found to be increasing compared to previous reviews, which was attributed to improved diagnostic testing. They also found that perioperative and long-term survival rates have improved, suggesting that many cases go undiagnosed and improved screening guidelines will highly likely improve outcomes^[21]^.

In a prospective registry study of 270 newly diagnosed ns-TAAD cases under the age of 60, 43% presented as acute aortic dissection. After screening relatives of these newly diagnosed probands, 216 new diagnoses were made, with 181 of these diagnoses found in FDRs. Of the 216 newly diagnosed relatives, 86 relatives were diagnosed through a sporadic proband, which transitioned these “sporadic” pedigrees to a familial aortopathy pedigree. In further support outlining the extent of phenotypic variability, six individuals who were initially clinically proven to be unaffected by an aortopathy went on to experience aortic dissection during the follow-up period. One in six patients under the age of 60 presenting with thoracic aortopathy met the author’s diagnostic criteria for ns-TAAD^[22]^. This highlights the increased incidence of heritable TAAD and thus a need for more defined screening and management guidelines for ns-TAAD.

Most recently, a retrospective/prospective study utilizing the GenTAC (Genetically Triggered Thoracic Aortic Aneurysm and Cardiovascular Conditions) Registry compared age and probability of elective surgery for thoracic aortic aneurysm, any dissection, and cardiovascular mortalities in patients with BAV aortopathy, MFS, LDS, vEDS, Turner syndrome, and ns-TAAD^[23]^. BAV had the highest ratio of elective ascending aortic replacement to emergent dissection surgery and the lowest risk of cardiovascular mortality. Interestingly, in this study, ns-TAAD patients experienced an aortic surgery (elective or emergent) at a more heterogenous age distribution and experienced dissection at a shorter time interval from diagnosis of their aortopathy compared to s-TAAD patients. This highlights the need for a better understanding of ns-TAAD to guide clinical management - diagnosis and timing of elective prophylactic repair.

Guidelines for MFS and BAV intervention and screening are notably better established, and a handful of studies have compared outcomes of ns-TAAD to those with MFS and BAV. A single-institution prospective surveillance study that sought to identify clinical and survival outcomes of patients with ns-TAAD in comparison to patients with MFS or BAV identified 311 patients out of 760 with a diagnosis of ns-TAAD^[24]^. Dissection at presentation and a family history of dissection were more common in ns-TAAD compared to BAV and MFS. Additionally, most fatal presentations due to aortopathy were in those with ns-TAAD (79.5%) compared to MFS (16.4%) and BAV (4.1%). Interestingly, they found that the natural history of ns-TAAD was comparable to the clinical course of an undiagnosed and untreated MFS. This posits the hypothesis that the late diagnosis of TAAD at the time of dissection in ns-TAAD patients is due to a lack of key extra-thoracic phenotypes that may trigger screening.

Sherrah *et al*. also found that a positive family history of dissection, younger age at diagnosis, and diagnosis of ns-TAAD or MFS were independent predictors of mortality^[24]^. As such, they advocate for management guidelines of ns-TAAD that are similar to guidelines for MFS. Kimura *et al*. support a similar management style. They conducted a multi-institutional retrospective review to determine outcomes of non-MFS acute aortic dissection and found similar rates in ns-TAAD and MFS aortic growth rates (0.23 cm/year *vs*. 0.19 cm/year, respectively), concluding that ns-TAAD patients be managed with guidelines comparable to those with MFS^[25]^. Their conclusions are in line with guidelines proposed by other groups, which support a more liberal threshold for elective repair of thoracic aortic aneurysm for ns-TAAD that mimics the lower aortic diameter thresholds for s-TAAD.

### Bicuspid aortopathy

As the most common congenital cardiac anomaly, BAV affects up to 2% of the general population and is commonly associated with thoracic aortopathy^[26]^. BAV is often an isolated condition and demonstrates variability in phenotypic expression in association with aortic valve dysfunction (stenosis and/or insufficiency) and TAAD. Familial clustering has been identified, but a specific genetic correlation has yet to be elucidated^[27]^. In addition to genetic variants, variability in presentation can be attributed to hemodynamic factors from bicuspid valve morphology^[28]^. However, the type of bicuspid morphology has not been correlated with a specific aortic aneurysm phenotype^[29,30]^. While BAV can be associated with syndromes (e.g., Turner syndrome), the majority of BAV cases are familial or sporadic. More than 80% of individuals with BAV have no attributable family history. Furthermore, patients who have an asymptomatic or minimally symptomatic BAV often go undiagnosed until presenting with dissection like those with ns-TAAD^[31]^. Several gene variants have been implicated in the development of both BAV and ns-TAAD: ACTA2, TGFBR2, NOTCH-1, MUC4, and ROBO4^[32–34]^. This supports the hypothesis that BAV disease is polygenic with epigenetic modification, as a myriad of environmental and hemodynamic factors influence the phenotypic expression of BAV morphology and the development of TAAD. Notable genetic mutations identified in both BAV and ns-TAAD are implicated in abnormal TGF- signaling and dysregulation of vascular smooth muscle cells (VSMC) predisposing to aneurysmal dilation^[32,35]^.

### Genetics

Multiple genes have been identified in the development of ns-TAAD. Unlike MFS and other connective tissue disorders, highly penetrant single-gene variants (i.e., Mendelian inheritance) are unlikely to be associated with certain clinical characteristics or hereditary patterns. Familial ns-TAAD is more likely to be associated with previously identified genes, but sporadic ns-TAAD has recently been found to result from pathogenic copy number variants and genetic polymorphisms. Currently, 11 genes have been validated to cause heritable thoracic aortic disease, and 17 genes have a putative hereditary association awaiting confirmational evidence (as of 2021)^[15]^. While some have only been identified in ns-TAAD, many others are connected with both ns-TAAD and syndromic thoracic aneurysm and dissection (s-TAAD)^[15,36]^, which again highlights the difficulties in understanding the pathogenesis of ns-TAAD. These genetic mutations result in dysregulation of TGF- signaling, inappropriate remodeling of extracellular matrix (ECM), and disruption of VSMC function^[37]^.

As many ns-TAAD are present in patients without pathognomonic syndromic features, the identification of pathogenic genes significantly impacts the diagnosis and management of ns-TAAD. The ClinGen Aortopathy Expert Panel reviewed 53 genes that have been found to have some association in hereditary TAAD and categorized them into the strength of association in thoracic aortopathy, with category A1 indicating “definitive” association and A2 indicating “strong” association. Variants in five genes (ACTA2, MYH11, MYLK, LOX, PRKG1) were categorized as A2 in association with ns-TAAD^[38,39]^. Several other genes, including FBN1, SMAD3, TGFB2, TGFBR1, and TGFBR2, have observed genome variants that are associated with both ns- and s-TAAD. A list of key genes discussed herein is listed in Table 1.

#### Gene mutations in vascular smooth muscle cells

The major responsibility of VSMCs is contraction in response to pulsatile blood flow to mitigate stretch and return the vessel to normal diameter. Contraction relies on -actin and -myosin cyclic interaction to maintain proper vascular tone. The following genomic pathways are important for the proper development of the -actin and -myosin contractile units. Mutations in these genes, some within the same pedigree, highlight the significant degree of phenotypic variance.

##### ACTA2:

Mutations in the *ACTA2* gene resulting in modification of VSMC -actin protein and improper filament assembly are responsible for 15% of familial ns-TAAD. Guo *et al*. conducted a study of a large family of over four generations demonstrating an autosomal dominant inheritance pattern of ns-TAAD with decreased penetrance but who were found to not have a known genetic mutation. Genome-wide linkage analysis identified an inherited missense mutation in *ACTA2* that results in a dominant-negative mechanism that decreases actin filament assembly. Further sequencing of an additional 97 unrelated but affected families identified 14 families with the *ACTA2* mutation. Interestingly, *ACTA2*-mutation carriers also presented with other vascular abnormalities, including livedo reticularis, iris oculae, and patent ductus arteriosus (PDA). Penetrance of TAAD was variable and did not increase with age^[40]^.

##### MYH11:

Multiple studies have found missense mutations in *MYH11*, a gene encoding VSMC-specific isoforms of myosin heavy chain proteins, to be associated with ns-TAAD in about 1% of cases^[37]^. Zhu *et al.* first identified *MYH11* mutations in one unrelated French and one American family presenting with TAAD. The mutations have a dominant-negative mechanism resulting in an abnormal C-terminal coiled-coil region of the myosin heavy chain specific to smooth muscle cells. Zhu demonstrated incomplete penetrance of TAAD in addition to members of both families carrying a diagnosis of PDA. Notably, asymptomatic carriers of the mutation in both families had normal aortic diameters but were found to have decreased aortic compliance and increased pulse wave velocity, suggesting early manifestations of aortopathy^[41]^. In other studies, multiple families have been found to harbor an *MYH11* mutation associating ns-TAAD and PDA^[39,42]^. However, Harakalova *et al.* identified two families with *MYH11* mutations that failed to segregate in the TAAD/PDA affected families, indicating the development of aortopathy is a multifactorial process with a role for environmental and epigenetic factors not only genetics^[43]^.

##### MYLK:

Two gain-of-function variants in *MYLK* (myosin light chain kinase) have been associated with the development of ns-TAAD. Upon sequencing of 193 probands, two families possessed different variants (one nonsense, four missense) in the *MYLK* gene but displayed a similar phenotype - aortic dissection with minimal to no previous aortic enlargement^[44]^. Additionally, Hannuksela *et al.* evaluated a five- generation family with genetic sequencing and aortic measurements. Their findings include five of the 19 living members experiencing aortic dissection or rupture, with only two surviving the event. Interestingly, those experiencing dissection or rupture had no differences in aortic stiffness or diameter compared to non-carriers of the mutation in the same family. These findings indicate that dissection in those who carry an *MYLK* mutation may not be preceded by a measurable, clinical aortic change^[45]^.

##### PRKG:

Exome sequencing identified a variant gain-of-function mutation in PRKG1, which encodes a type I cGMP-dependent protein kinase for signal transduction in VSMCs. The missense mutation in PRKG1 (c.530G>A) demonstrates complete penetrance in > 18 years of age and correlates with TAAD. Of the 31 family members carrying the PRKG1 gene, 63% presented with an acute aortic dissection. There was no gender difference in the age of dissection, and the average age of dissection for females and males was 31 years old. The mutation results in increased PKG-1 activity, leading to decreased VSMC contraction, although the subsequent mechanism leading to TAAD has yet to be elucidated^[46]^.

##### FOXE3:

Kuang *et al*. performed exome sequencing and identified pathologic variants of the *FOXE3* gene expressed in eight members of a large family presenting in an autosomal dominant pattern with incomplete penetrance. Mutations in the N-terminal end of *FOXE3*, a forkhead transcription factor, have been previously found in association with a spectrum of eye disorders^[47,48]^. However, none of the TAAD-affected individuals demonstrated eye abnormalities associated with a missense mutation in the C-terminal end of the DNA-binding domain (p.Asp153His). This novel mutation in *FOXE3* led to limited VSMC proliferation, differentiation, and survival in the ascending aorta and aortic arch during development, suggesting that *FOXE3* deficiency predisposes to ns-TAAD through heritable stress-induced VSMC apoptosis^[48]^.

##### MAT2A:

Whole-genome linkage analysis and exome sequencing of a large family with AD TAAD and BAV disease identified missense mutations in *MAT2A* (p.Glu344Ala, p.Arg356His), which encodes methionine adenosyltransferase II alpha, a critical enzyme in VSMC metabolism. Within one family of 18 members, 24% of those individuals were also found to have BAV. The study concluded that the risk of acute aortic dissection was low but still overall uncertain in the presence of BAV in these individuals^[37,49]^. These loss-of-function variants decrease enzyme function, leading to global hypomethylation that modulates phenotype and proliferation. The data suggests a potential epigenetic factor in the development of ns-TAAD.

#### Gene mutations in extracellular matrix

##### LOX:

Lysyl oxidases, including those encoded by the *LOX* gene, are extracellular copper enzymes that catalyze lysine cross-linking in elastin and collagen. Whole-genome and exome sequencing identified a heterozygous loss-of-function mutation in *LOX* in individuals with TAAD who present with some syndromic features of MFS (pectus deformities, striae, *etc*.) but fail to meet true MFS diagnostic criteria^[50,51]^. Several missense mutations in *LOX* result in decreased lysyl oxidase activity, which is proposed to result in fewer elastin cross-links and thus less organized, more fragmented elastic fibers within the thoracic aorta ECM that predisposes to TAAD. Interestingly, Guo *et al*. identified three of the 20 *LOX* mutation carriers also presented with a BAV, which posits the hypothesis that *LOX* defects may contribute to BAV disease and associated aortopathy^[51]^.

##### MFAP5:

Barbier *et al*. utilized whole-exome sequencing and mutation screening to identify a nonsense (p.Arg158*) and a missense (p.Trp21Leu) mutation in *MFAP5* in two families with ns-TAAD^[52]^. The gene encodes microfibril-associated glycoprotein 2 (MAGP-2), a component of the ECM found in fibrillin-containing and elastin-associated microfibrils. Both families presented with aortic root dilation without the other phenotypic features of s-TAAD. Aortic tissue collected at the time of surgery from one affected individual in the studied proband exhibited disorganization of the media with loss of VSMCs and proteoglycan accumulation on histology - typical features of TAAD. Barbier *et al*. propose that the loss-of-function of *MFAP5* disrupts ECM fibers, which affects the structural integrity of the aorta, leading to dilation, aneurysm formation, and dissection^[52]^.

##### THSD4:

Exome and next-generation sequencing of 35 families with TAAD and 1,114 unrelated TAAD patients, respectively, identified two heterozygous nonsense mutations in *THSD4*, which encodes the microfibril-associated protein ADAMTSL6^[53]^. This protein promotes fibrillin-1 matrix assembly, and these loss-of-function variants result in impaired assembly that correlates with progressive dilation of the thoracic aorta. Histologic staining of aortic samples from affected individuals demonstrates medial degeneration and diffuse ECM disruption.

##### FBN-1:

Perhaps the most well-known mutation in TAAD is FBN-1, which encodes fibrillin-1 and is the etiology of MFS. However, multiple studies have found that patients who presented with TAAD but did not meet the Ghent criteria for MFS also exhibit genetic variations at FBN-1^[54–57]^. These studies suggest that mutation of fibrillin-1 plays a role in the pathophysiology of ns-TAAD that is distinct from but not dissimilar to that of s-TAAD.

#### Gene mutations in TGF- signaling

##### TGFB2:

Most commonly associated with LDS^[58]^, loss-of-function mutations in *TGFB2* have also been found to cause ns-TAAD. Using genome-wide linkage analysis followed by exome sequencing in two large families with non-syndromic thoracic aortic disease, Boileau *et al*. identified four mutations in *TGFB2* causing haploinsufficiency in affected individuals^[59]^. The gene encodes transforming growth factor beta-2 (TGF-2), an important cell signaling molecule, and has paradoxically increased expression in diseased thoracic aortic tissue. The exact mechanism of this phenomenon has yet to be fully elucidated.

##### *TGFBR1* and *TGFBR2*:

Also underpinning LDS, *TGFBR1* and *TGFBR2* variants have been found in ns-TAAD. The genes encode two TGF-2 receptors involved in TGF-/Smad cell signaling. Pannu *et al*. identified *TGFBR2* mutations affecting Arg460 in the cytoplasmic serine-threonine kinase domain of the receptor in four of 80 families with ns-TAAD using exon sequencing^[60]^. Affected individuals presented with predominantly ascending aortic disease (aneurysm or Type A dissection); descending aortic and medium-vessel aortopathy was also present. Data from the Montalcino Aortic Consortium, an international registry of patients with heritable thoracic aortic disease, revealed ns-TAAD patients with a *TGFBR1* mutation have a greater aortic risk, particularly if they are female, but a *TGFBR2* mutation was associated with smaller aortic root diameter prior to or at the time of type A aortic dissection^[61]^.

##### SMAD3:

SMAD proteins play an integral role in downstream TGF- signaling in gene expression. Loss-of-function *SMAD3* mutations are strongly associated with LDS type 3 (aneurysms-osteoarthritis syndrome) but were found by Regalado *et al*. to also be responsible for 2% of ns-TAAD with a mean age at dissection of 42 years old^[62,63]^. Importantly, they also found that 19% of individuals carrying a variant in *SMAD3* experienced extra-thoracic disease - abdominal aortic aneurysm, iliac artery aneurysm, or intracranial aneurysm - which argues for screening for other aneurysmal sites in those carrying a *SMAD3* variant^[62]^.

##### SMAD4:

Smad4 is a central mediator protein in the canonical TBF- signaling pathway in implicated in ns-TAAD. Zhang *et al*. first described the role of Smad4 in aortic aneurysm pathogenesis using a conditional gene knockout mouse model^[64]^. Deletion of Smad4 in VSMCs led to the development of thoracic aortic aneurysm and subsequent death from aneurysm rupture in all affected mice, positing that Smad4 is integral to aortic wall homeostasis. In 2019, Duan *et al.* identified a loss-of-function *SMAD4* variant for the first time in humans with ns-TAAD. *SMAD4* is canonically associated with TAAD in individuals with a concurrent diagnosis of juvenile polyposis syndrome (JPS), hereditary hemorrhagic telangiectasia (HHT), or combined JPS-HHT^[65–67]^. This Smad4 heterozygous missense mutation (p.Arg97Leu) was newly identified in individuals with thoracic aortopathy but no diagnosis of JPS or HHT^[67]^. This mutation resulted in increased *SMAD4* ubiquitination and proteasome-mediated protein degradation that correlated to reduced contractile protein gene expression in VSMCs. Taken together, the data suggests a role for Smad4 mutation in predisposing for early expression of ns-TAAD in a mechanism likely mediated through VSMCs.

#### Gene mutations in cell development

##### NOTCH1:

Notch1 is a Type 1 transmembrane protein in the Notch signaling pathway that regulates interactions between physically adjacent cells and is integral in cell development, including cardiogenesis, valvulogenesis, and angiogenesis. Loss-of-function mutations in *NOTCH1* have been identified in pedigree and exon analyses in both familial and sporadic BAV patients^[68,69]^. McKellar *et al.* identified two novel missense mutations (A1243V and P1390T) in *NOTCH1* in 10.4% of patients with a BAV and thoracic aortic aneurysm that were not present in patients with a BAV and normal aorta or patients with a trileaflet aortic valve and thoracic aortic aneurysm^[70]^. This investigation highlights a role for NOTCH mutations in the pathogenesis of BAV aortopathy, but it was underpowered for statistical significance. Further research on whether these variants are gain-of-function *vs*. loss-of-function mutations has yet to be published.

## SCREENING AND MANAGEMENT GUIDELINES

The current guidelines for screening and management of known thoracic aortopathy primarily focus on syndromic TAAD. The most recent consensus guidelines in the U.S., published in the *Journal of the American College of Cardiology* in 2022, are a result of wide collaboration between medical, surgical, and radiological experts^[6]^. Additional comprehensive guidelines have been published by the Canadian Cardiovascular Society^[71]^ and the European Society of Cardiology^[72]^. These groups share similar recommendations in most areas, including advocating for surgical intervention with an aneurysm diameter between 4.5 and 5.0 cm and recommending screening in FDRs. Of note, the 2022 AHA/ACC guidelines recommend the timing of surgical intervention for ns-TAAD to be a shared decision between patient and surgeon (Class I) but also recommend prophylactic thoracic aortic repair when aortic diameter is greater than or equal to 5.0 cm in asymptomatic, low-risk individuals or greater than or equal to 4.5 cm in high-risk individuals. The differences between the American, Canadian, and European guidelines mostly pertain to details related to screening modality and frequency as well as specifics in genetic testing.

The 2022 AHA/ACC guidelines include a section on genetic aortopathies with recommendations for genetic testing and screening of family members for TAAD. Obtaining a multigenerational family history for TAAD, unexplained death, and aneurysms in patients with aortic root/ascending aortic aneurysms, or aortic dissection, as well as obtaining genetic testing to identify pathogenic variants are Class I recommendations^[6]^. At the time of writing of the 2022 guidelines, the HTAD genetic testing panels include 11 high-penetrance genes confirmed to be high risk for TAAD: *TBN1*, *LOX*, *COL3A1*, *TGFBR1*, *TGFBR2*, *SMAD3*, *TGFB2*, *ACTA2*, *MYH11*, *MYLK*, and *PRKG1*^[38]^. The authors recognize the plethora of yet unknown variants that predispose to TAAD and argue that families with a high incidence of TAAD undergo genetic screening to advance our understanding of ns-TAAD. These guidelines also recommend cascade screening - imaging family members with known/likely pathogenic variants to identify at-risk individuals with enlarging asymptomatic thoracic aortas. Repeat screening in negative individuals is recommended every five or ten years, depending on patient age.

An expert consensus from a large multidisciplinary team in The Netherlands similarly reviewed current recommendations and literature with the purpose of providing more detailed criteria for identifying high-risk individuals and a preferred screening modality and schedule^[73]^. They recommend genetic testing include the “core genes” implicated in ns-TAAD: *ACTA2*, *FBN1*, *MATA2A*, *MFAP5*, *MYH11*, *MYLK*, *PRKG1*, *SMAD3*, and *TGFBR2*. Familial screening should begin at 25 years of age or 10 years before the youngest case of complication in the family. Transthoracic echocardiography and CT or MRI axial imaging are the preferred screening modalities. Similar to the AHA/ACC guidelines, individuals lacking a genetic variant in ns-TAAD families with baseline normal screening should have repeat screening every five years.

A recent systematic review of 53 studies aimed to quantify the effectiveness of screening tests (via genetic testing and imaging) in relatives of both familial and sporadic ns-TAAD cases^[74]^. A total of 2,696 relatives underwent screening, including genetic testing in 49%, imaging studies in 11%, and a combination of genetic testing and imaging in the remaining 40%. First-, second-, and third-degree relatives were all included. Benefits of routine screening in both first- and second-degree relatives were indicated, with a possible benefit in third-degree relatives. They identified a total of 695 second-degree relatives who were screened and found 24% to be newly diagnosed compared to 33% of 893 first-degree relatives. This study supports screening for first- and second-degree relatives as well as the need for individualized screening, although they do recognize that diagnostic yield in various screening techniques is unknown. Moreover, Robertson *et al.* examined 216 relatives of newly diagnosed sporadic ns-TAAD cases (no known family history of TAAD)^[22]^. Screening of relatives from these probands yielded a new diagnosis in 86 additional relatives (40%), altering the hereditary pattern of diagnosed individuals from sporadic to familial ns-TAAD.

## FUTURE RESEARCH OPPORTUNITIES

Four overarching aims define areas of future research in ns-TAAD: (1) refining understanding of heritability patterns; (2) identifying novel pathogenic genes; (3) investigating the epigenetic contribution to aortic pathophysiology; and (4) individualizing screening and treatment algorithms. High-quality evidence in these areas is lacking, largely due to the phenotypic inconsistencies. However, recent strides have been made in verifying the importance of family history and screening for first and second- degree relatives. To further investigate heritability risk and genetics, whole population studies will be beneficial. The majority of published literature on these topics is from a few specialized centers and is subject to referral bias. A whole population study focused on delineating heritability patterns would include those individuals who are unaware they are at risk or aware of risk but choose not to pursue treatment, thereby decreasing the risk of selection bias. Additionally, broadening the inclusion net to a whole population sample could identify new heritability patterns and provide a better foundation for future genetic research in genetic linkage analyses and genome-wide association studies to discover the impact of significant polygenic and epigenetic factors. Stratifying familial risk of inheritance and genetic factors will advance developments in individualized screening algorithms.

## CONCLUSION

Thoracic aortic dissection is a feared complication of aortic aneurysm due to its high mortality rates, but it is preventable through prophylactic surgical intervention. With advances in genomics and the emergence of a personalized medicine paradigm for the diagnosis and treatment of aortic disease, there is a greater focus on understanding the biochemical underpinnings of ns-TAAD to better inform patient care. The purpose of this review article is to summarize the foundation of knowledge in ns-TAAD heritability and outline recent genetic research developments. Non-syndromic individuals are at particular risk for dissection due to the difficulty of diagnosing asymptomatic aneurysms without associated clinical features as well as variable penetrance and phenotypic expression of mutations that lack classical Mendelian inheritance patterns. This complexity has made the development of screening and intervention guidelines for ns-TAAD challenging. Current guidelines emphasize the importance of genetic testing, serial imaging, and prophylactic thoracic aortic replacement for individuals in high-risk ns-TAAD families. However, these guidelines still lack the specificity present for s-TAAD and other cardiac diseases, convoluting the recommended shared decision making between surgeons and patients. Avenues of future research involve risk stratification of relatives based on family history, characterization of genetic involvement, discovery of new pathogenic genes, and individualization of screening algorithms.

## Figures and Tables

**Table 1.** Key genes associated with ns-TAAD

	Protein encoded	Type of mutation	Key features

**Mutations in VSMCs**			
*ACTA2*	-actin	Dominant-negative missense	Associated with livedo reticularis, iris oculae, PDA
*MYH11*	Myosin heavy chain proteins	Dominant-negative missense	Associated with PDA
*MYLK*	Myosin light chain kinase	Gain-of-function nonsense, missense	Present with aortic dissection with minimal/no aortic enlargement
*PRKG*	Type I cGMP-dependent protein kinase	Gain-of-function missense	Correlates with acute aortic dissection
*FOXE3*	Forkhead transcription factor	Loss-of-function missense	Not associated with eye abnormalities
*MAT2A*	Methionine adenosyltransferase II alpha	Loss-of-function missense	Associated with BAV aortopathy
**Mutations in ECM**			
*LOX*	Lysyl oxidase	Loss-of-function missense	Associated with pectus deformities, striae, BAV
*MFAP5*	Microfibril-associated glycoprotein 2	Loss-of-function nonsense, missense	Associated with aortic root dilation
*THSD4*	Microfibril-associated protein ADAMTSL6	Loss-of-function nonsense	Impaired fibrillin-1 matrix assembly correlates with progressive thoracic aorta dilation
*FBN-1*	Fibrillin-1	Loss-of-function missense, nonsense, deletion	Etiology of MFS, also present in ns-TAAD
**Mutations in TGF-signaling**			
*TGFB2*	Transforming growth factor beta-2	Loss-of-function nonsense, missense, deletions	Etiology of LDS; paradoxically increased expression levels found in diseased thoracic aortas
*TGFBR1/TGFBR2*	Transforming growth factor beta-2 receptors	Loss-of-function or dominant-negative missense	Associated with LDS; predominantly ascending aortic disease (aneurysm, dissection)
*SMAD3*	Smad3 signal transduction protein	Loss-of-function missense	Associated with LDS type 3 and ns-TAAD presenting with extra-thoracic aneurysms
*SMAD4*	Smad4 signal transduction protein	Loss-of-function missense	Can be associated with JPS, HHT, or combined JPS-HHT
**Mutations in cell development**			
*NOTCH1*	Notch1 transmembrane protein	Loss-of-function missense	Associated with aortic valve development and BAV

VSMCs: Vascular smooth muscle cells; PDA: patent ductus arteriosus; BAV: bicuspid aortic valve; ECM: extracellular matrix; MFS: marfan syndrome; ns-TAAD: non-syndromic thoracic aortic aneurysm and dissection; LDS: loeys-Dietz syndrome; JPS: juvenile polyposis syndrome; HHT: hereditary hemorrhagic telangiectasia.

## Data Availability

Data and materials are available by request to the contributing author.
